# Bidirectional effects between maternal mental health and adolescent internalizing problems across six years in Northern Ireland

**DOI:** 10.1002/jcv2.12078

**Published:** 2022-05-31

**Authors:** Justin M. Luningham, Bethany Wentz, Christine E. Merrilees, Laura K. Taylor, Marcie C. Goeke‐Morey, Peter Shirlow, Tess Shannon, E. Mark Cummings

**Affiliations:** ^1^ Department of Biostatistics & Epidemiology, School of Public Health University of North Texas Health Science Center Fort Worth Texas USA; ^2^ Department of Psychology University of Notre Dame Notre Dame Indiana USA; ^3^ Psychology Department State University of New York Geneseo Geneseo New York USA; ^4^ School of Psychology University College Dublin Dublin Ireland; ^5^ School of Psychology Queen’s University Belfast Belfast UK; ^6^ Department of Psychology Catholic University of America District of Columbia USA; ^7^ Institute of Irish Studies University of Liverpool Liverpool Merseyside UK

**Keywords:** adversity, family functioning, internalising disorder, longitudinal studies, maternal factors

## Abstract

**Background:**

Emerging evidence indicates the existence of bidirectional relations between mothers’ mental health and adolescent adjustment, but few studies have examined these relations in contexts of high environmental adversity, including economic deprivation and political violence. Given other empirical connections between political violence and adolescent adjustment problems, the impact of child adjustment problems on maternal mental health may be exacerbated in contexts of sectarian violence.

**Methods:**

Addressing this gap, latent change score modeling was used to examine interrelations between trajectories of maternal mental health and adolescent internalizing symptoms over time in communities afflicted by political conflict. Over six years, 999 adolescent‐mother dyads participated in a longitudinal study in Belfast, Northern Ireland. Six‐hundred ninety‐five families were originally recruited in year 1, with 304 recruited to supplement the sample in year 3; the largest available sample for a given year was 760 dyads. Models including maternal mental health, adolescent internalizing symptomatology, and political violence (i.e., sectarian antisocial behavior) as a time‐varying covariate were tested.

**Results:**

Results demonstrated that for both mothers and adolescents in a dyadic pairing, higher rates of symptomology in one member of the dyad were related to symptoms observed in the other member. Results also suggest that political violence and factors related to social deprivation increased symptoms across the dyad.

**Conclusion:**

This study advances understanding of the bidirectional impact between maternal mental health and adolescent internalizing over time in contexts of political violence.

## Graphical abstract

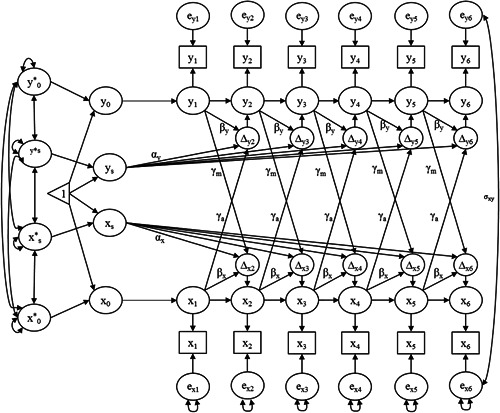

This study examined the bidirectional impact between maternal mental health and adolescent internalizing 6 consecutive years in youth from Belfast, Northern Ireland. Results demonstrated that higher symptomology in one pair of the mother‐adolescent dyad predicted an increase of symptoms for the other member in the following year. Politically‐related antisocial behavior in the community increased mother's and adolescent's symptomatology, thereby impacting these bidirectional relations.

**Key points**

- Further exploring known correlations between maternal mental health and adolescent internalizing psychopathology, this study examined the bidirectionality of maternal and adolescent functioning within communities experiencing sociopolitical adversity
- Bidirectional relations were analyzed over six consecutive years in families living in communities impacted by political violence in Belfast, Northern Ireland
- Results demonstrated that higher symptomology in one pair of the mother‐adolescent dyad predicted an increase of symptoms for the other member in the following year. Politically‐related antisocial behavior in the community increased mother’s and adolescent’s symptomatology, thereby impacting these bidirectional relations
- Findings suggest that community programs designed to improve adolescent adjustment in adverse community contexts could benefit from involving the entire family system

The impact of maternal mental health on child development has been a major area of research for many years. Empirical work suggests a bidirectional relationship between maternal mental health and child adjustment, indicating that child functioning also impacts maternal functioning (Cummings & Schermerhorn, 2003). However, little is known about factors that may influence these relations in high‐risk contexts, such as contexts of socioeconomic deprivation and political violence. Recent research has examined interrelations between maternal and adolescent mental health in contexts of socioeconomic deprivation (Baker et al., 2019; Milan & Carlone, 2018; Shaw et al., 2016), but questions remain about dynamic change in the maternal‐adolescent relationship over extended time periods in communities experiencing political violence. Currently, there is limited evidence to confirm that a bidirectional effect persists in adverse contexts. Given other empirical connections between political violence and a range of adolescent adjustment problems (Cummings et al., 2017), the impact of child adjustment problems on maternal mental health may be more pronounced in such conditions.

In the current study, we examined bidirectional relations between maternal mental health and adolescent internalizing symptomatology in the context of socioeconomic deprivation and political violence in Northern Ireland. We hypothesized that maternal mental health and adolescent internalizing symptomatology are predictive of each other’s prospective longitudinal trajectories. We also hypothesized that the context of political violence specifically would impact maternal mental health and child internalizing, with higher perceived conflict in the community increasing symptomology for each pair in this reciprocal dyad. Better understanding of these effects over time may lead to improved intervention strategies for mothers and adolescents in high‐risk communities (Cummings et al., 2017).

## Bidirectionality between maternal and adolescent mental health

Past research has established that maternal depression predicts child internalizing symptomatology and indicates a possible reciprocal relationship in internalizing symptomatology between mothers and children (Goodman & Gotlib, 1999; Goodman et al., 2011). For example, Elgar et al. (2003) examined reciprocal effects of children’s adjustment problems and mothers’ depressive symptomatology. Their four‐year longitudinal study of 16,581 parent‐child dyads found a bidirectional risk of child adjustment problems and maternal depression at clinically significant levels. Specifically, children with depressed mothers were twice as likely to engage in problematic behaviors compared to their peers, and maternal depression also increased with more reported emotional problems in children. Mothers’ and children’s distress were thus dynamically interrelated over time, even after prior levels of distress were statistically controlled. Recently, Spiro‐Levitt et al. (2019) analyzed the relationship between mother‐child depressive symptomatology in 167 dyads undergoing a Depression Prevention Initiative (DPI) program. A significant positive correlation between child improvement in depressive symptoms and maternal improvement in depressive symptoms was found; as the adolescents improved over the two years, the mothers did as well. Sellers et al. (2016) was one of the first longitudinal studies to establish that adolescent depressive symptoms could trigger a subsequent recurrence of maternal depressive episodes in high‐risk mothers who had been previously diagnosed with major depressive disorder.

## Bidirectional effects in contexts of socioeconomic inequality and political violence

Recent research has focused on relationships between maternal and adolescent mental health in specific contexts of adversity, including socioeconomic deprivation. For example, Shaw et al. (2016) conducted two longitudinal studies examining the links among child conduct problems, maternal depression, and neighborhood deprivation. In their first study, using mother‐son dyads in an economically deprived urban area, longitudinal effects of child conduct problems on maternal depression and maternal depression on childhood conduct problems were indicated, supporting a reciprocal relationship between childhood externalizing symptomatology and maternal depression. Their second study validated the predictive effects of maternal depression on childhood conduct problems. Neighborhood deprivation was predictive of conduct problems in children in both studies. In the current study, we hypothesized that indicators of political violence, commonly associated with socioeconomic deprivation, would factor into maternal‐adolescent relations similarly.

Furthermore, Baker et al. (2019) examined the bidirectional relationship between child behavioral problems and maternal depression using longitudinal cross‐lagged modeling in mother‐child dyads in low‐income families. Mothers reported on their own depressive symptomatology, their parenting, and their child’s internalizing and externalizing symptomatology. Bidirectional relationships were found between behavioral problems in children and maternal depression across every wave of the study.

Milan and Carlone (2018) explored how depression and PTSD symptoms in mothers and adolescent daughters predicted their relational behaviors among mother‐daughter dyads from diverse, low‐income backgrounds. Adolescents’ depressive symptoms significantly impacted their mothers’ behaviors towards them after accounting for maternal symptoms. While mothers’ relational behaviors predicted changes in adolescents’ depressive symptoms, adolescent behaviors were unrelated to subsequent maternal symptoms. Mother and daughter PTSD symptoms both predicted daughters’ relational behaviors.

Current work thus supports bidirectional relations between maternal and child mental health in communities with lower socioeconomic statuses. However, bidirectional relations may differ widely depending on the context, and little is known about relations in other contexts adversities, such as ethnopolitical conflict. Given links between political violence and adolescent adjustment problems (Cummings et al., 2017), it is expected that internalizing problems for adolescents will impact maternal mental health symptoms and that reciprocal relations will be found. The current study breaks new ground by examining relations between adolescent internalizing and maternal mental health in a multi‐wave longitudinal study of families in Northern Ireland in communities afflicted by protracted political violence.

## Northern Ireland

The present study utilizes data from families with adolescents in socially deprived neighborhoods in Belfast, Northern Ireland, where families face socioeconomic adversity and exposure to political conflict. Data were collected between 2006 and 2012, a period of time that provided a unique setting for studying the effects of post‐accord political conflict on youth’s adjustment. Although the roots of the conflict can be traced back for centuries, the most recent concerns focus on the 30‐year period of violent conflict known as the Troubles (1968‐1998) and its aftermath, in which Nationalists/Republicans (typically Catholics) contended for unification with the Republic of Ireland and Unionists/Loyalists (typically Protestants) to remain part of the United Kingdom. The 1998 Belfast Agreement formally resolved the Troubles, yet sectarian conflict continues to play a role in Northern Ireland. Many neighborhoods and schools in Belfast remained segregated by ethnicity, and there were thousands of sectarian‐motivated crimes and incidents reported to Northern Ireland police annually during the period of study (Police Service of Northern Ireland, 2019).

The current study sampled from families with preadolescent and adolescent youth, as this demographic is more likely than others to be exposed to political conflict and more likely to actively contribute over time to intergenerational transmission of political violence (McEvoy‐Levy, 2006). Adolescents in Northern Ireland around the time of data collection were not merely passive victims of sectarianism, but were active participants in protests, rioting, and acts of violence (McEvoy‐Levy, 2006; Northern Ireland Riots, 2011). Moreover, as paramilitary organizations in Northern Ireland were disbanded, evidence indicates that adolescents were more likely than other demographics to be involved in sectarian conflicts; young people, unaffiliated with such organizations, demonstrated increased activity in antisocial behavior (e.g., rioting and crime; Haydon & Scraton, 2008; Shirlow & McEvoy, 2008).

Negative outcomes for youth associated with political violence and armed conflict have been identified worldwide (Cummings et al., 2017). Additionally, research has identified links between exposure to political violence, family processes, and psychopathology in adolescence. Family processes are longitudinal predictors of youth adjustment in contexts of political violence (Betancourt et al., 2013; 2015; Panter‐Brick et al., 2011). Further examination of relations between environmental adversity, family processes, and adolescent adjustment is warranted to improve understanding of family functioning in contexts of political violence.

## The present study

In the current study, trajectories of maternal mental health and adolescent internalizing symptoms were modeled in 999 adolescent and mother dyads who participated in a six‐year longitudinal survey study in Belfast, Northern Ireland. We hypothesized that symptomology in mothers and adolescents would be reciprocally and positively associated with each other, accounting for political violence in each family’s local community. We also hypothesized that political violence would further increase symptoms and reciprocal relations in the dyad.

## METHOD

### Participants

The data come from a 6‐year longitudinal survey of mother‐adolescent dyads in Belfast, Northern Ireland. Mothers and one adolescent were surveyed annually. For households with more than one child, the youngest child eligible for participating was selected to maximize the amount of time families could be followed before the child left home. About half of participating adolescents had older siblings; others were the eldest or only children. At the start of data collection, 695 dyads were surveyed; a supplemental sample was added at time 3 for a total of 999 unique family dyads across the six years. Mothers were surveyed instead of fathers. Mothers head many households in working‐class Belfast, and generally were more available for in‐home surveys. The sample was exclusively White, consistent with the Northern Irish population (98.2% White at last Census, NISRA, 2012). At time 1, adolescent ages ranged from 8‐15 (*M* = 12.13). Table 1 provides mean and age ranges for all 6 years of study. The adolescent gender distribution for the sample was approximately 48% male and 52% female. Families were selected using stratified random sampling. An expert demographer on Belfast’s ethnic composition informed the selection of 35 families from specific wards (i.e., neighborhoods), all of which were high in social deprivation according to Northern Ireland government statistics (Cummings et al., 2010). Neighborhood ethnic groupings were homogeneous (at least 90% protestant or 90% Catholic). The sample was representative of the overall population in Belfast (43% Catholic, 57% Protestant; Darby, 2001).

**TABLE 1:** Descriptive statistics of the variables used in the study reported as “mean (standard deviation)”

	Year 1	Year 2	Year 3	Year 4	Year 5	Year 6
**Internalizing**	4.75 (3.19)	4.20 (2.93)	4.49 (2.78)	4.39 (2.83)	3.91 (2.75)	3.42 (2.17)
**GHQ**	11.06 (5.29)	10.65 (5.74)	13.82 (5.90)	12.42 (6.07)	10.88 (6.27)	10.02 (5.51)
**Child SAB**	2.99 (5.74)	2.83 (6.85)	5.25 (9.13)	2.93 (6.92)	2.90 (6.84)	2.16 (5.31)
**Mom SAB**	2.60 (6.01)	2.06 (5.83)	3.86 (8.07)	1.64 (5.56)	1.85 (5.76)	1.00 (3.93)
**Age [min, max]**	12.13 [8, 15]	13.20 [9, 16]	13.63 [10, 17]	14.70 [11, 18]	15.79 [12, 19]	16.87 [13, 20]
**N (prev. year attrition)**	695 (0)	567 (0.18)	760 (0.19)*	622 (0.18)	586 (0.06)	583 (0.01)

*Note*: The age range for each year is reported instead of SD. Attrition is reported as the proportion of lost families for a given year relative to the previous year. The asterisk* for year 3 denotes that a supplemental sample of 304 families was added at this time; attrition is based on the continuation of the original 567 from year 2 to year 3.

Abbreviations: GHQ, general health questionnaire; SAB, sectarian antisocial behavior.

### Procedure

Families were surveyed in the late spring from 2006 to 2012. In‐home, in‐person interviews were conducted by an established survey company based in Northern Ireland with considerable experience working in the Belfast community. Interviewers were accredited under the Interviewer Quality Control Scheme and registered under the Data Protection Act. Interviewers surveyed mothers and adolescents separately over approximately 90 minutes. Research protocol and measures were approved by all Institutional Review Boards at all participating universities. Families participating in the study were given monetary compensation for their time. Families received £20 for participation at times 1 and 2, £40 at times 3 and 4, and £50 at times 5 and 6.

### Measures

**Adolescent internalizing symptoms.** Adolescents completed the Strengths and Difficulties Questionnaire (SDQ), including subscales for emotion problems and peer problems (Goodman, 1997). Goodman et al. (2010) recommend combining the emotion and peer subscales to assess internalizing symptomology in non‐clinical populations. Adolescents responded to each item with 0 (*not true*), 1 (*somewhat true*), or 2 (*certainly true*). Example items include “I am often unhappy, down‐hearted, or tearful” and “Other children or young people pick on me or bully me.” On average, internal consistencies for internalizing scores were 0.62.

**Maternal mental health.** Mothers completed the 12‐item version of the General Health Questionnaire (GHQ‐12), which measures mental health indicators (Goldberg et al., 1997). Responders rate their mood and level of distress on a scale from 1 (*not at all* or *much less than usual)* to 4 (*much more than usual)*. Sample items include “Have you recently lost much sleep over worry?” and “have you recently felt that you are playing a useful part in things?” Higher scores indicate more mental distress. On average, internal consistencies for maternal mental health were 0.89.

**Sectarian Antisocial Behavior.** Adolescents and mothers completed the Sectarian Antisocial Behavior Scale (SAB) to assess awareness of these behaviors within their communities. The SAB is a 12‐item scale assessing respondents’ awareness in the previous 3 months to a range of sectarian‐motivated behavior, from witnessing stones thrown over peace walls to politically‐driven murder. Items were answered using a 5‐point Likert‐type scale, with choices ranging from (1) *(not in the last 3 months)* to (5) (*every day)*. Average Cronbach’s alphas were 0.94 (mother report) and 0.93 (child report).

### Statistical analysis

Univariate and bivariate latent change score models (LCSMs) were used to characterize longitudinal trajectories (McArdle & Hamagami, 2001, McArdle, 2009). The LCSM was chosen because it allows for explicitly modeling the change that occurs from one time point to the next, as opposed to modeling the score of the outcome at a specific point in time. These models accommodate both difference score and time series concepts into models of change and are more dynamic than linear growth curves (McArdle, 2009). Importantly, LCSMs can accommodate pairs of variables and provide interpretable tests of relationships between their trajectories. With mother‐adolescent dyads, the LCSM can test if maternal scores at time *t* directly influence the change in adolescent symptoms between time *t* and time *t* + 1, and vice versa. The analysis proceeded in two steps. First, the best LCSM was determined to characterize trajectories of adolescent internalizing symptoms and maternal mental health separately. Second, the trajectories were modeled simultaneously to test for dyadic effects between mothers and adolescents. All latent variable models were fitted using the *lavaan* package (Rosseel, 2012) in the *R* statistical computing software (R Core Team, 2021). We assumed the data were missing at random for families that did not respond in a given year and estimated the model using full‐information maximum likelihood to address missing data.

**FIGURE 1:**
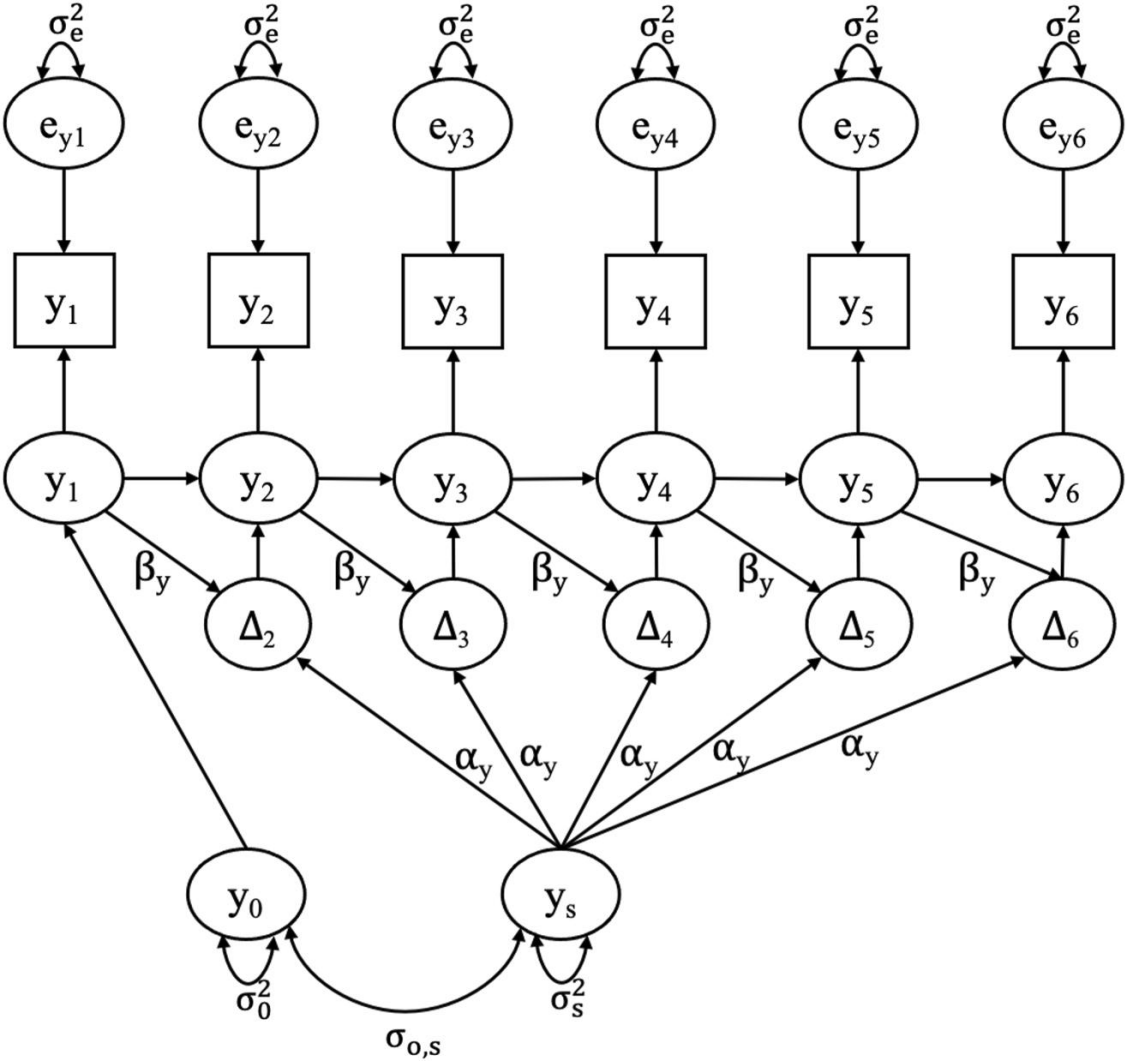
Path diagram of a 6‐time point latent change score model. y_0_ represents a latent intercept, y_s_ represents a latent slope, e_y_ represents residual variance of the observations, *α* represents the constant change over time (i.e., linear slope), *β* represents the proportional change over time (i.e., autoregressive change), and *Δ* represents the latent change factor from one year to the next. The latent change factors are freely estimated; thus change is not strictly linear, but can be dynamic. Unlabeled paths are fixed to 1.

**Univariate longitudinal models.** Univariate LCSMs were tested to evaluate the trajectory of adolescent and maternal distress symptoms separately. The LCSM includes a constant change parameter, which indicates that the processes are changing linearly (with intercept and slope parameters) and a proportional change parameter that allows for dynamic, possibly non‐linear growth. Proportional change means that the rate of change depends on the initial severity of symptoms. A path diagram representing univariate LCSM is presented in Figure 1. Sequential models were fitted examining linear change only, proportional change only, and the dual‐change LCSM with both change processes. SAB was included as a time‐varying covariate in all models (i.e., adolescent’s GHQ score at time 1 was regressed on adolescent’s SAB score at time 1). The effect of SAB was freely estimated at each time point because the context of political conflict changed year‐to‐year.

**FIGURE 2:**
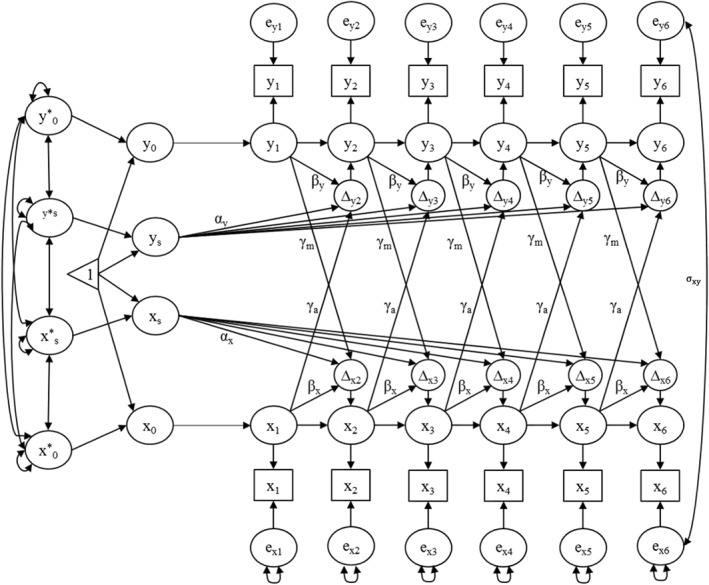
Full bivariate latent change score model. Change processes modeled in Figure 1 are simultaneously modeled for maternal and adolescent symptoms. Dyadic coupling is modeled by *γ_a_* and *γ_m_* (impact of adolescent internalizing at time *t* on the change in maternal mental health distress between time *t* and *t* + 1, and vice versa), plus correlations between residuals x = adolescent internalizing, y = maternal general health questionnaire.

**Dyadic longitudinal models.** Next, the bivariate LCSM was fitted (Figure 2). In this model, the best‐fitting trajectories for adolescents and mothers from the univariate step were fitted simultaneously, and different patterns of “coupling” were tested across the dyad. Direct coupling effects included the impact of adolescent internalizing at time *t* on the latent change factor for mothers’ mental health distress at time *t* + 1 *and* the impact of maternal distress at time *t* on adolescent outcomes at time *t* + 1. The coupling parameter *γ* was set to be equal across time points. Covariances were estimated between the latent intercepts and slopes. Residual covariances between mothers and adolescents were also included within each time point and constrained equal over time, as is conventional for dual LCS models (McArdle, 2009). To account for the effects of ethnopolitical conflict, adolescents’ and mothers’ scores on the SAB scale were included as time‐varying predictors of internalizing and GHQ scores at each time point. The time‐varying nature of SAB was important to allow for years with unique sectarian‐related events. We also controlled for age by regressing adolescent intercept and slope on each person’s age at baseline.^1^

Models were compared using a *χ* ^2^ difference test, comparing nested models that differed by inclusion or exclusion of key parameter estimates of interest. If a simpler model did not fit significantly worse than its more complex counterpart, then the simpler model was preferred over the fuller model because the additional parameter(s) did not significantly improve fit with the observed data. In these analyses, the full model included dyadic coupling, and reduced models allowed for only maternal impact on adolescents, only adolescent impact on mothers, and no coupling in the dynamic process.

## RESULTS

Table 1 includes descriptive statistics for GHQ, internalizing, SAB, and child age across all 6 years. This table also presents sample size at each year and year‐to‐year attrition.

Figure S1 provides a 24 x 24 correlation matrix of internalizing, GHQ, mother’s SAB, and child SAB across all 6 years to show longitudinal associations. Univariate results, presented in Table 2, indicated that dual LCSM was the most appropriate trajectory for characterizing maternal mental health symptoms and adolescent internalizing symptoms, based on *χ* ^2^ model fits and information criteria. In other words, simplifying the change trajectory for either mothers or adolescents by removing proportional or linear change resulted in significant deterioration to the model fit, supporting the dual change model for each.

**TABLE 2:** Univariate change models for maternal general health questionnaire (GHQ) and adolescent internalizing scores

Maternal GHQ scores			
	*df*	*χ* ^*2*^	RMSEA
Constant change	51	403.103	0.084
Proportional change	53	826.52	0.122
Dual Change*	50	390.83	0.083

Adolescent Internalizing			
	*df*	*χ* ^*2*^	RMSEA
Constant change	55	324.41	0.071
Proportional change	58	753.55	0.110
Dual Change*	54	281.54	0.065

Abbreviations: df, degrees of freedom; RMSEA, root mean square error of approximation.

The bivariate model comparison results are presented in Table 3. Starting with the fully coupled process model, the model displayed good fit, *χ* ^2^(211) = 831.12, p < 0.001; RMSEA 90% confidence interval (CI) = [0.051, 0.059]; Standardized Root Mean Square (SRMR) = 0.079. Removing the direct effect of maternal mental health on adolescent latent change resulted in a significantly worse model (*Δ χ* ^2^ (1) = 8.45, p = 0.004). Removing the effect of adolescent symptoms on maternal symptoms resulted in even more significant change in model fit (*Δ χ* ^2^(1) = 21.08, *p <* 0.001). Removing both dynamic coupling parameters also resulted in a much worse model fit, as did removing any dyadic connection through the intercept and slope covariances. The results thus supported a model in which symptoms for one pair of the dyad directly impacted the amount of change year‐over‐year for the other partner in the dyad and removing any point of connection between the two partners resulted in a worse‐fitting model. A model that constrained the effect of SAB to be time‐invariant for adolescents and mothers fit significantly worse as well, *Δχ* ^2^(10) = 32.36, p < 0.001, suggesting that the impact of SAB fluctuated each year, depending on political events unfolding in Belfast.

**TABLE 3:** Summary of Goodness‐of‐fit comparisons for bivariate latent change score models

*Model*	*df*	*χ* ^*2*^	∆*df*	∆*χ* ^*2*^	SRMR	90% RMSEA
Complete bivariate coupling	211	831.12	‐‐	‐‐	0.079	.051 ‐ .059
Internalizing → GHQ only, *γ*_m_ = 0	212	839.57	1	8.45, p = .004	0.082	.051 ‐ .059
GHQ → Internalizing only, *γ*_a_ = 0	212	852.19	1	21.08, p < .001	0.081	.052 ‐ .059
No coupling relations, γ_m_ = *γ*_a_ = 0	213	860.67	2	29.557, p < .001	0.084	.052 ‐ .060
No connection (uncorrelated slopes, level, and residuals)	218	953.29	7	122.17, p < .001	0.094	.055 ‐ .062

Abbreviations: df, degrees of freedom; GHQ, maternal general health questionnaire; RMSEA, root mean square error of approximation; SRMR, standardized root mean squared residual.

Key parameter estimates are presented in Table 4. Critically, the coupling parameter estimates were statistically significant and positive in value, meaning higher symptoms in one pair for each year contributed to higher symptoms in the other member for the following year. The proportional change coefficients were negative in value and remained significant, but the linear trajectory was no longer significant when accounting for the coupling process. In other words, changing symptoms from year‐to‐year were a function of the previous year’s symptoms (in addition to the coupling effect) and depended upon one’s initial level (i.e., intercept). While both mothers and adolescents generally declined in symptoms by the final year of study, higher symptomatology of the other partner in the dyad could slow or even reverse this decline in symptoms. Figure S2 displays model‐predicted trajectories of internalizing and GHQ scores over the six years for mothers and adolescents with high, average, and low scores. Adolescent internalizing at time *t* had a larger impact on maternal mental health at time *t* + 1 than did the maternal proportional decline coefficient (0.635 (SE = 0.14) vs. −0.343 (SE = 0.08), respectively). Ultimately, mothers with high initial GHQ symptoms and whose adolescents displayed higher levels of internalizing realized an initial increase in GHQ score over time, before returning to initial levels in later years (Figure S2).

**TABLE 4:** Parameter estimates from the best‐fitting model and accompanying interpretation. Refer to Figure 2 for graphical display of the model

Parameter	Est (SE)	*p* value	Interpretation
*β_x_*	−0.748 (0.09)	<0.001	Proportional change score, internalizing
*β_y_*	−0.343 (0.08)	<0.001	Proportional change score, GHQ
*γ_a_*	0.635 (0.14)	<0.001	Prediction of adolescent internalizing at time *t* on the proportional growth of maternal GHQ at time *t* + 1
*γ_m_*	0.154 (0.06)	0.006	Prediction of maternal GHQ at time *t* on the proportional growth of adolescent internalizing at time *t* + 1
Mean of *x*_0_	5.73 (0.90)	<0.001	Internalizing intercept score
*α_x_*	0.942 (0.65)	0.146	Internalizing linear slope score
Mean of *y_0_*	11.40 (0.25)	<0.001	GHQ intercept score
*α_y_*	0.996 (1.00)	0.320	GHQ linear slope score
*σ_xy_*	1.161 (0.22)	<0.001	Residual covariance between mother and adolescent within time‐point
**Time‐varying SAB effects**			
Time	mSAB → GHQ	aSAB → INT	
1	0.049 (0.03)	−0.046 (0.02)*	
2	0.137 (0.04)**	0.121 (0.02)***	
3	0.0147 (0.03)***	0.045 (0.01)***	
4	0.082 (0.04)*	0.021 (0.01)	
5	0.044 (0.04)	0.013 (0.01)	
6	−0.088 (0.06)	0.02 (0.02)	

Abbreviation: aSAB, adolescent‐rated sectarian antisocial behavior; GHQ, general health questionnaire; mSAB, mother‐rated sectarian antisocial behvarior; SE, standard error.

*, *p* < 0.05; ***, *p* < 0.00.

It was also critical to examine the effect of SAB at each time point, allowing for time‐varying effects to reflect differences from year to year in sectarian conflict. For both mothers and adolescents, SAB had a significant positive prediction of symptoms at times 2 and 3 (Table 4). SAB also had a significant positive effect on GHQ at time 4, and had a small, surprising negative effect on internalizing at time 1. The correlation between mother and adolescent SAB report was high within each wave of the study (Figure S1). Based on the model, those with unusually high SAB would result in symptom increases for a member of the dyad, which would impact the other through the coupling process. Finally, residual covariance within each time‐point was positive and significant between mothers and adolescents in the model. This result indicates that there were additional sources of connection reflecting the socioecological context between mother and adolescent mental health near the time of data collection.

## DISCUSSION

The present study examined the bidirectional relationship between mothers’ mental health and adolescent internalizing symptomatology, controlling for the context of political violence in each family’s local community. The findings indicated that maternal mental health and adolescent internalizing symptoms display dynamic, bidirectional effects on each other over time. Within the dyad, each partner’s symptomatology prospectively predicted the other’s symptoms. The results also suggested that political violence and other factors related to social deprivation impacted these relations. Specifically, both mothers and adolescents generally declined in symptomatology temporally; this decline occurred in proportion to the level of symptomatology expressed when they entered the study. However, the rate of decline was slowed or reversed by the symptomatology of the other partner in the dyad. Adolescent internalizing at time *t* had a larger impact on maternal mental health at time *t* + 1 than did the maternal proportional decline coefficient, thus highlighting the critical effect adolescent functioning has on maternal functioning over time. Moreover, it was found that high initial levels of adolescent symptomology resulted in a net increase in mental health problems (i.e., GHQ scores) for mothers over time.

These findings support past research which emphasizes the reciprocal relationship between maternal and child depressive symptomatology (Baker et al., 2019; Elgar et al., 2003; Hughes & Gullone, 2010). Our results further this work by demonstrating that mothers’ and adolescents’ symptomatology was prospectively predictive of the other’s symptoms, even after accounting for the context of ethnopolitical conflict. In fact, SAB and factors related to social deprivation generally increased each member of the dyad’s symptoms, therefore impacting the other’s trajectory over time.

### Limitations

This study has a few limitations to consider. First, these results are based entirely on self‐reported questionnaire assessments. Another limitation is that we operationalized emotional difficulties as continuous traits. Although this methodology yields some strengths (e.g., continuous outcome scores increase statistical power and differentiate the severity of problems among non‐clinical samples in the absence of formal diagnosis), these results may not apply to those with clinical diagnoses. Additionally, there were low internal consistencies in the SDQ scale. However, the SDQ is widely used, and relatively low internal consistencies are typically reported in other studies as well. Finally, this study considers only the relations between mothers and adolescents and did not survey fathers. The role of fathers' impact on adolescent adjustment in Northern Ireland is discussed elsewhere (Luningham et al., 2020).

### Future directions

Future research should address the impact of bidirectional effects between adolescent internalizing symptomatology and paternal mental health. Past work (Cummings et al., 2016) supports that emotional security in the family or in the community merits consideration in models testing the bidirectional link between maternal and adolescent functioning.

## CONCLUSIONS

Our study emphasizes the significance of the bidirectional relationship between maternal functioning and adolescent adjustment, and the exacerbation of these relations in contexts of political violence. This study suggests that family processes, specifically the relationship between mothers’ mental health and adolescent internalizing symptomatology are not only linked but influence each other over time.

## AUTHOR CONTRIBUTIONS

**Justin M. Luningham:** Conceptualization, Formal analysis, Investigation, Methodology, Visualization, Writing – original draft, Writing – review & editing. **Bethany Wentz:** Investigation, Methodology, Validation, Writing – original draft, Writing – review & editing. **Christine E. Merrilees:** Conceptualization, Data curation, Funding acquisition, Investigation, Supervision, Writing – review & editing. **Laura K. Taylor:** Conceptualization, Data curation, Funding acquisition, Project administration, Validation, Writing – review & editing. **Marcie Goeke‐Morey:** Conceptualization, Data curation, Funding acquisition, Project administration, Writing – review & editing. **Peter Shirlow:** Data curation, Funding acquisition, Project administration, Writing – review & editing. **Tess Shannon:** Investigation, Writing – original draft, Writing – review & editing. **E. Mark Cummings:** Conceptualization, Data curation, Funding acquisition, Investigation, Resources, Supervision, Validation, Writing – original draft, Writing – review & editing.

## CONFLICT OF INTEREST

The authors have declared that they have no competing or potential conflicts of interest.

## ETHICAL CONSIDERATION

Research protocol and measures were approved by Institutional Review Boards at all participating universities. Mothers provided consent for their own participation and consent for their child's participation.

## Supporting information

Figure S1

## Data Availability

The data that support the findings of this study are available on request from the corresponding author. The data are not publicly available due to privacy or ethical restrictions.

## ENDNOTE

1 For those who entered the study in year 3 as part of the supplemental sample, age at baseline was calculated as age at year 3 minus 2.
