# Supplementary material for: Bidirectional effects between maternal mental health and adolescent internalizing problems across six years in Northern Ireland
Source: JCPP Adv. 2022 May 31;2(2):e12078. doi: 10.1002/jcv2.12078 (PMC9815047; doi:10.1002/jcv2.12078)
Supplement: Supplementary file 1 — Figure S1 [file JCV2-2-e12078-s001.docx]

**
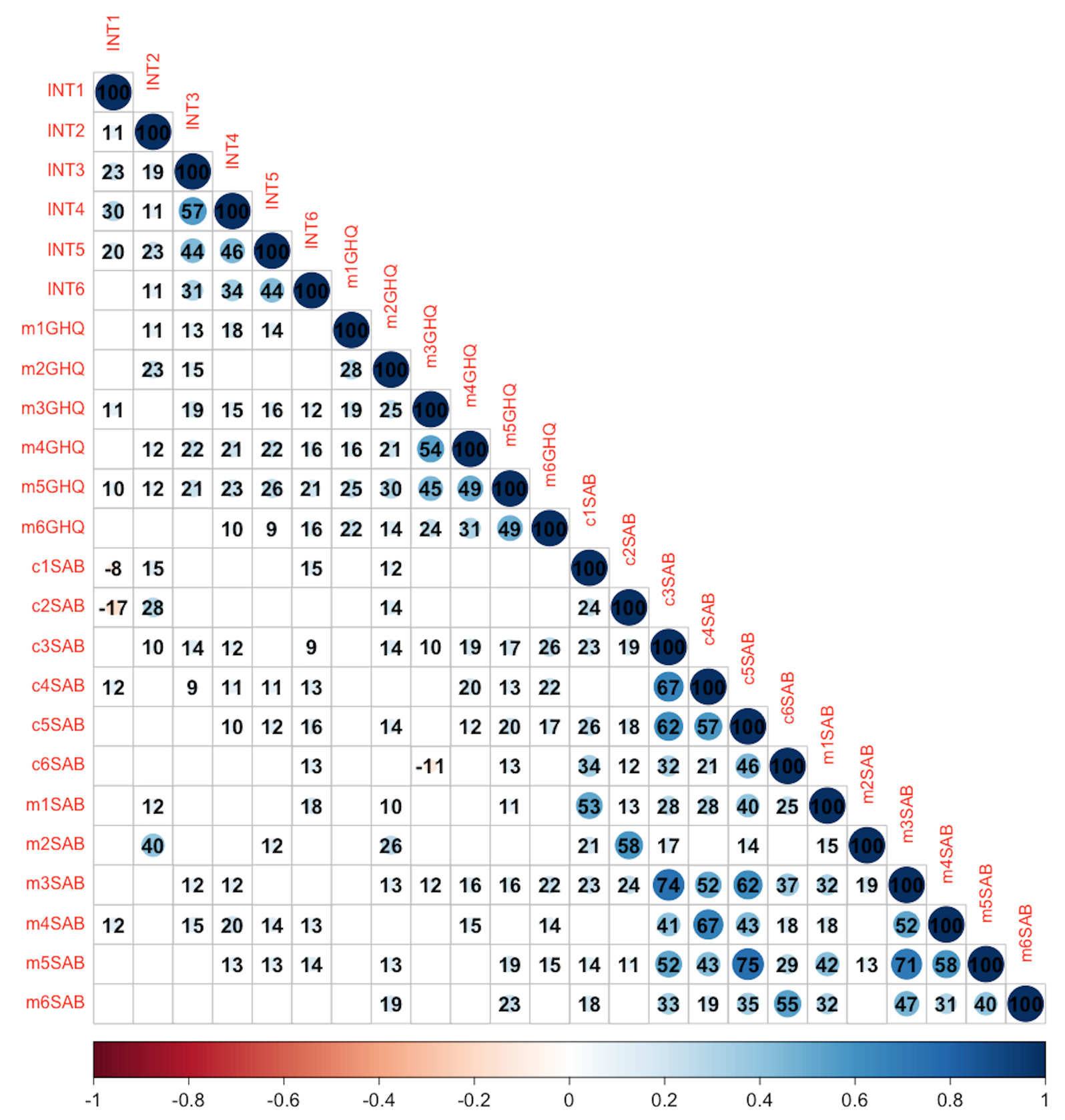
 Figure S1.** Correlation matrix of internalizing, GHQ, and SAB measures across six years of study. Numbers represent correlation coefficients as percentages. Numbers presented are significant at the p < 0.05 level; empty cells are non-significant. GHQ, general health questionnaire; SAB, sectarian antisocial behavior.

**
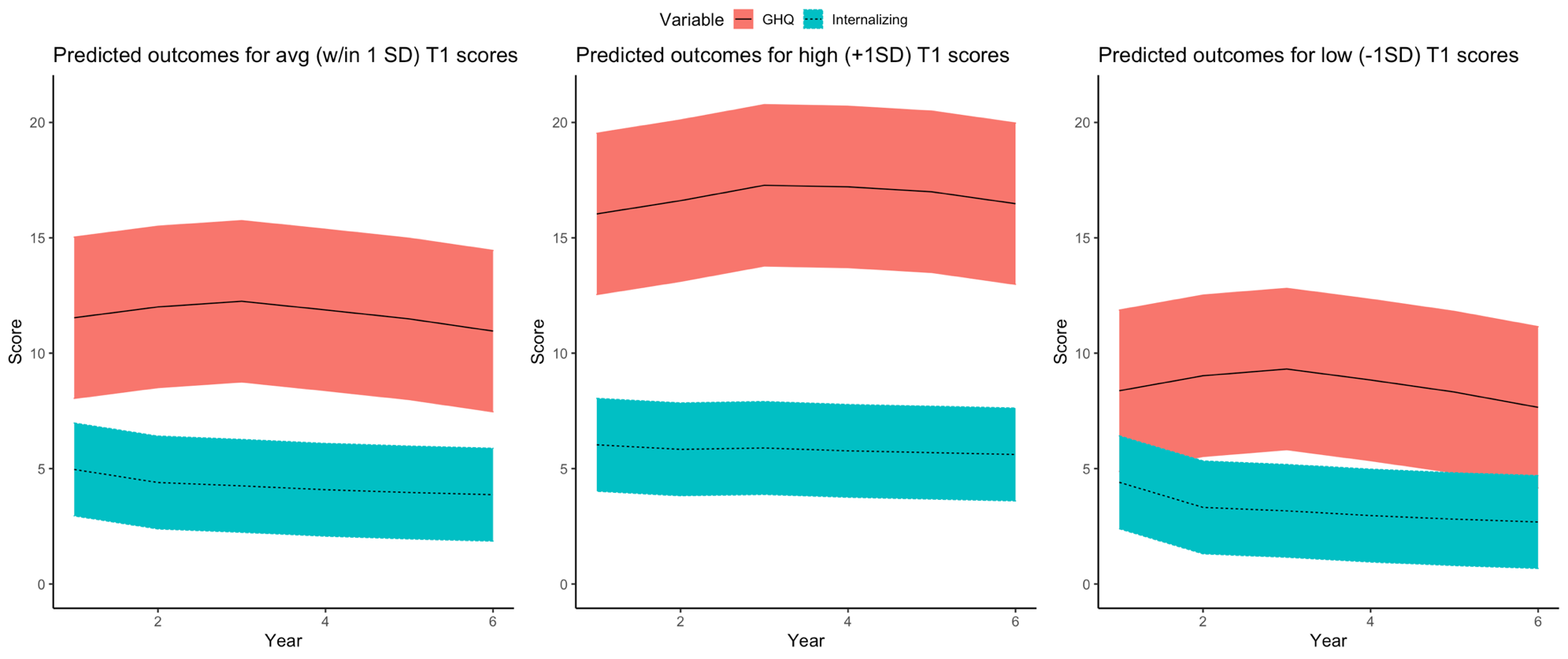
**

**Figure S2.** Average predicted values over time and prediction intervals. Interval ranges computed using within-person and between-person variability. Families were stratified based on predicted GHQ scores at time 1 being greater than +1 SD beyond the mean, less than -1SD from the mean, or within 1 SD of the mean. GHQ was used as the criteria to keep mother-adolescent pairs joined together, rather than selecting adolescents who were high on internalizing but from different families. The three panels demonstrate the differences in trajectories due to the proportional change component, in which the values at time *t* are a function of the score at time *t – 1.* High initial levels of GHQ (middle panel) correspond to an initial increase in maternal GHQ symptoms over the first three years and a slower decline in internalizing symptoms for the matched adolescent. For average families (left panel), mothers display a slight increase in symptoms initially before a small decline, and internalizing symptoms generally decline. For families with low initial levels (right panel), mothers still experience a small increase in the first couple of years, followed by another decline, and the overall symptom levels remain low. For these families, internalizing also starts low and exhibits a slightly quicker decline than the other groups.
